# Antimicrobial Prophylaxis for Recurrent Urinary Tract Infections in Premenopausal and Postmenopausal Women: A Retrospective Observational Study from an Outpatient Clinic in a Tertiary University Hospital

**DOI:** 10.3390/antibiotics14100998

**Published:** 2025-10-05

**Authors:** Tomislava Skuhala, Marin Rimac, Vladimir Trkulja, Snjezana Zidovec-Lepej

**Affiliations:** 1 Department for Urogenital Infections, University Hospital for Infectious Diseases “Dr. Fran Mihaljević”, 10000 Zagreb, Croatia; tskuhala@bfm.hr; 2 School of Dental Medicine, University of Zagreb, 10000 Zagreb, Croatia; 3 Zagreb University School of Medicine, 10000 Zagreb, Croatia; 4 Department of Pharmacology, Zagreb University School of Medicine, 10000 Zagreb, Croatia; vladimir.trkulja@mef.hr

**Keywords:** recurrent urinary tract infections, antimicrobial prophylaxis, premenopausal women, postmenopausal women, retrospective study

## Abstract

**Background:** Recurrent urinary tract infections (rUTIs) significantly impair women’s quality of life, making antimicrobial prophylaxis a critical preventative strategy. This retrospective observational study aimed to characterize antibiotic prophylaxis patterns, relapse rates, comparative efficacy of different agents, and tolerability in 908 women (663 postmenopausal, 245 premenopausal) with rUTIs managed at a tertiary university hospital. **Methods:** Data from medical records (January 2022–December 2024) were analyzed. Patients were stratified by menopausal status. We assessed antibiotic usage, relapse rates (per 100 patient-months), and adverse events. Comparative efficacy of nitrofurantoin-based versus fosfomycin/other prophylaxis was evaluated for rUTIs caused by *E. coli*, *E. faecalis*, or *E. coli* ESBL using weighted and matched analyses to control for covariates. **Results:** Continuous antimicrobial prophylaxis was the primary strategy, with nitrofurantoin being most frequently used. Premenopausal women showed a greater tendency for intermittent or combined prophylactic approaches. Postmenopausal women exhibited a higher overall crude relapse rate (5.54/100 p-m) compared to premenopausal women (3.14/100 p-m), with *E. coli* being the most common causative agent in relapses. For rUTIs caused by *E. coli*, *E. faecalis*, or *E. coli* ESBL, nitrofurantoin-based prophylaxis demonstrated significantly lower adjusted relapse rates than fosfomycin/other regimens (rate ratio: 0.47 for postmenopausal, 0.35 for premenopausal women). This observed efficacy for nitrofurantoin was robust against potential unmeasured confounding. Prophylaxis was generally well-tolerated (3.0% gastrointestinal adverse events overall); however, premenopausal women reported a higher adverse event incidence. **Conclusions:** Our findings strongly suggest that nitrofurantoin is an effective prophylactic choice for rUTIs caused by common uropathogens (*E. coli*, *E. faecalis*, *E. coli* ESBL), particularly in postmenopausal women. The diverse prophylactic strategies highlight the need for individualized care. While generally well-tolerated, adverse event profiles vary between menopausal groups, necessitating careful monitoring.

## 1. Introduction

### 1.1. Epidemiology and Definition

Recurrent urinary tract infections (rUTIs) are common among women of all ages. Approximately 60% of women experience at least one episode of cystitis during their lifetime [1]. It is estimated that 20–40% of women who experience one episode of cystitis will likely experience another, and 25–50% of them are expected to have recurrent infections [2,3].

rUTIs are defined as the occurrence of two or more episodes of UTIs within six months or three or more episodes within one year that are confirmed by urine culture. Infections and reinfections can occur in patients with or without risk factors. These risk factors include, but are not limited to, anatomical or functional abnormalities of the urinary tract, post-void residual urine, urine incontinence, neuro-urological conditions, immunocompromised states, pregnancy, pelvic organ prolapse, recent antibiotic use, indwelling urinary catheters, recent urological instrumentation, the presence of urinary stones, urinary obstruction, and resistant microorganisms as causative agents [4,5].

### 1.2. Predisposing Factors

The risk factors for rUTIs are consistent with those for isolated UTIs and they differ based on age and menopausal status [6,7]. In premenopausal women, the most significant risk factor is recent sexual intercourse, followed by the use of spermicides [8,9,10,11]. Additional risk factors include a new sexual partner within the past year, first UTI onset before the age of 15, and a positive family history. Although other factors have been proposed (such as urination habits in relation to intercourse, urinary frequency, delayed voiding, post-defecation hygiene practices, various showering and bathing habits, wearing tight-fitting underwear, and body mass index) their association with rUTIs has not been confirmed [9].

In postmenopausal women estrogen deficiency leads to atrophic vaginitis and alterations in the vaginal microflora, both of which compromise the integrity of the vaginal epithelium and facilitate vaginal introitus colonization by uropathogens. Additional risk factors in this population include urinary incontinence, cystocele, and incomplete bladder emptying [6,12].

The use of urinary catheters and functional deterioration (both physical and cognitive), particularly in institutionalized elderly women, further increases the risk of UTIs [12,13,14].

### 1.3. Causative Agents

The most common causative pathogen in rUTIs is *Escherichia coli* (*E. coli*). In 52–77% of cases, the same *E. coli* strain is responsible for both the initial and recurrent infections [15,16]. The risk of recurrence is higher if the initial episode was caused by *E. coli* compared to infections caused by other bacterial species [17,18].

Other bacterial pathogens in rUTI include Klebsiella pneumoniae (*K. pneumoniae*), Proteus mirabilis (*P. mirabilis*), Enterococcus faecalis (*E. faecalis*), and Staphylococcus saprophyticus (*S. saprophyticus*), whose prevalence varies by patient group and can be associated with anatomical abnormalities, catheter use, or antibiotic exposure [19].

### 1.4. Antimicrobial Prophylaxis

Women with rUTIs experience significant reductions in quality of life due to chronic physical symptoms, psychological distress, and negative effects on social and sexual wellbeing. Studies also highlight an increase in anxiety, depression, and disruption of daily activities [20,21,22].

The prevention of rUTIs plays an important role in enhancing the quality of life of affected women. Various preventive strategies can be used (non-antimicrobial and antimicrobial prophylaxis) with differing degrees of efficacy, guided by evidence-based recommendations issued by professional societies which regularly update clinical guidelines.

Antimicrobial prophylaxis is considered the most effective method for rUTI prevention [23,24,25] and it is strongly recommended by all relevant professional societies [4,5,26,27,28,29]. When antimicrobial prophylaxis is indicated, careful patient selection is essential and multiple factors such as comorbid conditions, ongoing chronic therapies, sensitivity of uropathogens to antibiotics, and the potential for adverse drug reactions must be considered before initiating therapy.

Due to the potential adverse effect of antibiotics (side effects and interactions with other drugs, development of bacterial resistance, clostridial diarrhea, effect on intestinal and vaginal flora), antimicrobial prophylaxis should be considered if non-antimicrobial measures were not effective.

There are three types of antimicrobial prophylaxis of rUTIs [4,5,28,29]:

- Continuous low-dose prophylaxis—consists of administration of 1/4 or 1/2 of the therapeutic dose of antibiotics once a day before bedtime.
- Postcoital prophylaxis—consists of administering a single dose of an antibiotic selected based on previous urine cultures within two hours after sexual intercourse.
- Self-diagnosis and self-treatment—appropriate in patients with good compliance.

There is no significant difference in effectiveness between continuous antimicrobial prophylaxis (CAP) and postcoital prophylaxis in women with sexual intercourse as a risk factor for rUTI [4,5].

The optimal duration of CAP is not well defined but reported treatment durations range from 3 to 12 months. After discontinuation, recurrences are common, especially in women with three or more infections annually.

For antimicrobial prophylaxis, different antimicrobials can be used, and the choice should be guided by urine culture. Commonly recommended antibiotics are nitrofurantoin, fosfomycin, sulfamethoxazole–trimethoprim (SMX/TMP), cephalexin, cephaclor, and nitroxoline [4,5,30,31,32,33,34,35,36,37].

Knowledge gaps in antimicrobial prophylaxis of rUTIs still exist. In this context, we aimed to analyze the characteristics of female patients with rUTIs, and the use of antimicrobial prophylaxis specifically in peri-/postmenopausal and premenopausal women.

## 2. Results

### 2.1. Eligible Patients and Characteristics of the Index UTI Episode

**Figure 1:**
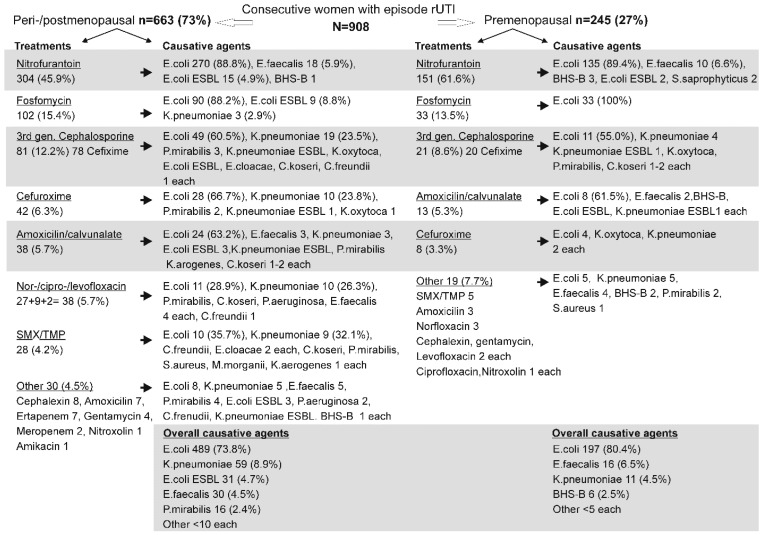
Characteristics of the index (r)UTI episode in peri-/postmenopausal and premenopausal women. Data for each of these two patient subsets are organized by most common treatment (in a descending order) and corresponding causative agents based on urine culture. The shaded area at the bottom sums up the most prevalent causative agents across all treatment options.

The present analysis embraces a total of 908 adult women with rUTIs who were managed for an acute UTI (index) episode, and were subsequently followed-up over a (varying) period of prophylactic treatment between 1 January 2022 and 31 December 2024 at the Outpatient Clinic for Urogenital Infections at the University Hospital for Infectious Diseases “Dr. Fran Mihaljević”, Zagreb Croatia (with a total of 2375 individual consultations). Of those, 663 (73.0%) were postmenopausal (managed through 1800 visits) and 245 (27.0%) were premenopausal women (managed through 575 visits). As summarized in Figure 1, the most common treatment of the index episode in both subsets was nitrofurantoin (45.9% of the post- and in 61.6% of the premenopausal women), followed by fosfomycin (15.4% and 13.5%, respectively), third-generation cephalosporines (12.2% and 8.6%, respectively); cefuroxime (6.3% and 3.3%, respectively); amoxicillin–clavulanate (5.7% and 5.3, respectively). The causative agents treated with these antibiotics were similar in the two subsets (Figure 1): *E. coli* was the most common one in all of these treatment options, but other causative agents somewhat differed between treatments, similarly in the post- and the premenopausal women.

Fluoroquinolones and sulfamethoxazole–trimethoprim (SMS/TMP) were somewhat more commonly used in postmenopausal [38 (5.7%) and 28 (4.2%), respectively] than in premenopausal women (only a few cases) (Figure 1). A number of other antibiotics were used in a smaller number of patients in both subsets (Figure 1). Overall, *E. coli* was the main causative agent in both patient subsets (73.8% and 80.4%, respectively) (Figure 1). Most other agents (*K. pneumoniae*, *E. coli* ESBL, *E. faecalis*) were similarly infrequently observed in the post- and premenopausal women, but there were also smaller differences regarding individual agents (Figure 1).

### 2.2. General Prophylactic Strategies in Peri-/Postmenopausal and Premenopausal Women

**Figure 2:**
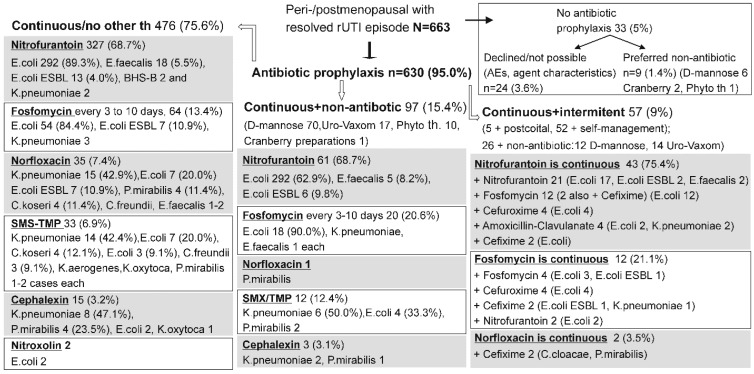
General prophylactic strategies employed after the resolution of the index (r)UTI episode in peri-/postmenopausal women. Three observed modes of treatment are illustrated: continuous antibiotic without any additional therapy (left), continuous antibiotic + non-antibiotic treatment (middle), continuous antibiotic + additional intermittent use (postcoital or self-management) (right). Within each mode, data are organized with respect to the most commonly used antibiotic (in descending order) chosen in line with the causative agents identified in the index episode (these agents are depicted for each antibiotic). Phyto th—various phytotherapeutic preparations.

Figure 2 summarizes the general prophylactic strategies employed in peri-/postmenopausal women. The resolution of the index episode was followed by a continuous antibiotic prophylaxis in 630/663 (95%) postmenopausal women, whereas 33 (5%) received no prophylaxis (24, 3.6%—either declined it, or a reasonable prophylaxis could not be designed given the microbiological findings), or opted for non-antibiotic treatments (9, 1.4%—mostly D-mannose) (Figure 2). Three different modes of continuous prophylaxis were employed: (i) only antibiotic with no additional treatments (in 476 or 75.6% women) chosen with respect to the causative agent identified in the index episode—typically nitrofurantoin (68.7%), less commonly fosfomycin (13.4%), norfloxacin (7.4%), SMS/TMP (6.9%), cephalexin (3.2%), and nitroxoline (two women) (Figure 2); (ii) continuous prophylaxis combined with non-antibiotic treatment (mostly D-mannose) (Figure 2) (in 97 or 15.4% women)—again with predominance of nitrofurantoin (68.7%), followed by fosfomycin (20.6%), SMS/TMP (12.4%), cephalexin (3.1), and norfloxacin in one woman (Figure 2); (iii) continuous prophylaxis later on “enhanced” by additional intermittent antibiotic use, either postcoital (rarely) or self-management (in 57 or 9% women) (Figure 2). Half of these women also used additional non-antibiotic treatments, D-mannose or Uro-Vaxom) (Figure 2). The most common continuously used antibiotic was again nitrofurantoin (75.4%) which was combined with additional nitrofurantoin or less commonly with other antibiotics (Figure 2), followed by fosfomycin (21.1%) combined with other antibiotics and norfloxacin (two women) (Figure 2).

Postmenopausal women managed by these three main modes of (continuous antibiotic) prophylaxis were of similar age and somewhat differed in prevalence of individual known risk factors, and their total number (Table 1). Overall, median duration of prophylaxis was 6 months (range 0.25–36 months) which generated 4011 patient-months of observation (Table 1).

The duration of the monitored prophylaxis was generally similar for all three treatment modes, but since they differed in the number of patients, different numbers of patient-months (p-m) were generated (Table 1). Overall crude relapse rate was 5.54 relapses/100 p-m of prophylactic exposure and was generally similar across the three treatment modes: 5.43/100 p-m for those with (continuous) antibiotic only, 7.20/100 p-m for women with additional non-antibiotic treatment, and 3.91/100 p-m in women with combined continuous and intermittent treatment (Table 1). The most common causative agents at the relapse episodes were practically identical to those observed in the index episode and were similar across the three treatment modes (Table 1).

**Figure 3:**
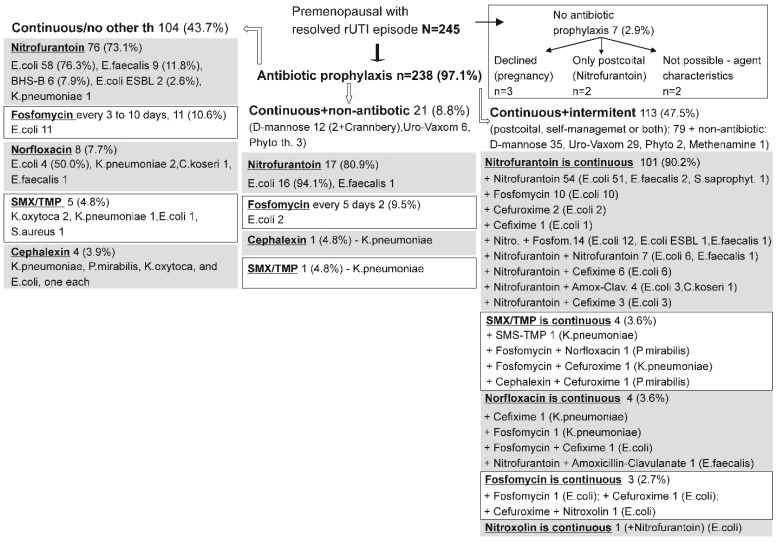
General prophylactic strategies employed after the resolution of the index (r)UTI episode in premenopausal women. Three observed modes of treatment are illustrated: continuous antibiotic without any additional therapy (left), continuous antibiotic + non-antibiotic treatment (middle), continuous antibiotic + additional intermittent use (postcoital or self-management, or both) (right). Within each mode, data are organized with respect to the most commonly used antibiotic (in descending order) chosen in line with the causative agents identified in the index episode (these agents are depicted for each antibiotic). Phyto—various phytotherapeutic preparations.

Figure 3 summarizes the general prophylactic strategies employed in premenopausal women. Of them, 238/245 (97.1%) received some form of continuous antibiotic prophylaxis, whereas seven either declined it (three women), or it was not possible (two women), or opted for only postcoital prophylaxis with nitrofurantoin (two women) (Figure 3). Again, three modes of prophylaxis were practiced, but with somewhat different frequencies as compared to postmenopausal women (depicted in Figure 2): (i) only antibiotic with no additional treatment (in 104 or 43.7% women) [mainly nitrofurantoin (73.1%), followed by fosfomycin (10.6%), norfloxacin (7.7%), SMS/TMP (7.7%), and cephalexin (3.9%); (ii) antibiotic + non-antibiotic (mainly D-mannose) (in 21 or 8.8% women) (nitrofurantoin in 17 women) (Figure 3); (iii) continuous treatment enhanced by additional postcoital or self-management use, or both, commonly combined with non-antibiotic treatment (manly D-mannose and Uro-Vaxom^®^) (Figure 3). Here, nitrofurantoin was also predominant as the main antibiotic, combined with additional nitrofurantoin, or less commonly with other drugs (Figure 3), whereas SMS/TMP-based, norfloxacin-based, fosfomycin-based, and nitroxoline-based treatments were sporadic (Figure 3).

Premenopausal women managed by these three main modes of (continuous antibiotic) prophylaxis were of similar age and somewhat differed in the prevalence of individual-known risk factors (Table 2) which, however, were far less common than in postmenopausal women depicted in Table 1. Overall, median duration of prophylaxis was 6 months (range 0.5–36 months), which generated 1337 patient-months of observation (Table 2). The duration of the monitored prophylaxis was generally similar for all three treatment modes, but since they differed in the number of patients, different numbers of patient-months (p-m) were generated (Table 2). Overall crude relapse rate was 3.14 relapses/100 p-m of prophylactic exposure and was generally similar across the three treatment modes: 3.96/100 p-m for those with (continuous) antibiotic only, 3.33/100 p-m for women with additional non-antibiotic treatment, and 2.33/100 p-m in women with combined continuous and intermittent treatment (Table 2). The most common causative agents at the relapse episodes were practically identical to those observed in the index episode and were similar across the three treatment modes (Table 2).

For both the post- and premenopausal women (Figure 2 and Figure 3) different prophylactic modes largely differed in their prevalence, with some treatment subsets being very limited in size, and with a number of very variable elements (causative agents, preferred “main” antibiotic) (Table 1 and Table 2), which precluded a meaningful comparison between different “modes”; however, it appears fair to state that both in post- and premenopausal women, relapse rates across the three “general prophylactic modes” seemed fairly similar.

### 2.3. Relative Efficacy of Nitrofurantoin-Based vs. Other Antibiotic-Based Prophylaxis

**Figure 4:**
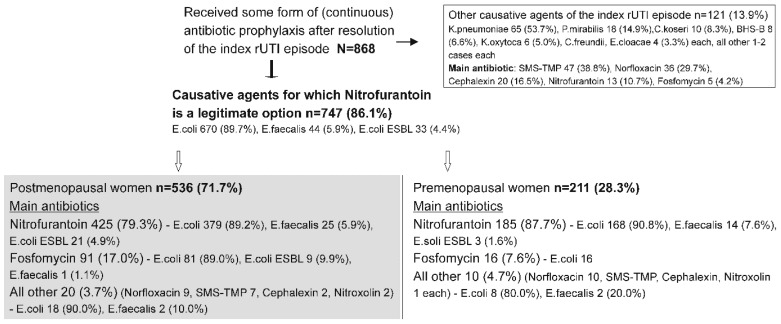
Characteristics of women in whom the index (r)UTI episode was caused by *E. coli*, or *E. faecalis*, or *E. coli* ESBL; nitrofurantoin, fosfomycin, and several other treatments are feasible prophylactic options.

Of the 868 women (in total) who received some form of continuous antibiotic prophylaxis, 747 (86.1%) suffered the index episode caused by either *E. coli* (89.7%), *E. faecalis* (5.9%) or *E. coli* ESBL (4.4%) (Figure 4)—agents for which nitrofurantoin was a feasible prophylactic option. All other causative agents of the index episode were cumulatively observed in 121 (13.9%) women, and their prophylaxis was based predominantly on agents other than nitrofurantoin (Figure 4).

Of the 747 women with the index episode caused with these three causative agents, 536 (71.7%) were postmenopausal, and 211 (28.3) were premenopausal (Figure 4). Among the former, 425 (79.3%) received nitrofurantoin-based prophylaxis, 91 (17.0%) received fosfomycin-based prophylaxis, and the remaining 20 (3.7%) received prophylaxis based on other compounds (Figure 4). Of the premenopausal women, 185 (87.7%) received nitrofurantoin-based prophylaxis, 16 (7.6%) received fosfomycin-based treatment, and the remaining 10 (4.7%) received prophylaxis based on other compounds (Figure 4).

The fact of common causative agents but different prophylactic treatments provided an opportunity to compare prophylactic efficacy of nitrofurantoin-based vs. fosfomycin- or other antibiotic-based prophylaxis, separately in post- and in premenopausal women. In each subset (post- or premenopausal), women receiving nitrofurantoin-based or “other antibiotic”-based prophylaxis were similar in age, prevalence or index-episode causative agents, but differed somewhat in prevalence of the risk factors, and “general modes” of treatment (continuous only, continuous + non-antibiotic, continuous + intermittent) (Table 3).

Duration of prophylaxis appeared slightly longer in nitrofurantoin-based treatments (median 6 months both in post- and premenopausal women) vs. other antibiotic-based treatments (median 4 months in both patient subsets) (Table 3). Crude relapse rates appeared lower for nitrofurantoin-based vs. other prophylaxis, both in postmenopausal (4.35 vs. 10.2/100 p-m), and in premenopausal women (2.36 vs. 5.31/100 p-m) (Table 3). Using optimization-based weighting (postmenopausal women) or exact matching (premenopausal women), those receiving nitrofurantoin-based and women receiving other compounds-based prophylaxis were perfectly balanced on the important covariates (Table 4): in postmenopausal women, adjusted relapse rates were clearly lower with nitrofurantoin-bases vs. comparator prophylaxis (rate ratio 0.47, 95%CI 0.34–0.67); the same trend was observed in premenopausal women (rate ratio 0.35, 95%CI 0.08–1.46), but the numbers of patients and relapses were low, so the estimate was imprecise (wide CIs) (Table 4). The E-value for the rate ratio in postmenopausal women (3.46) indicates that a set of hypothetical confounding variables would need to be 3.46 more prevalent in the nitrofurantoin-treated women vs. comparator women and would need to have a strong prophylactic effect (RR = 0.29, i.e., more then three-fold reduction in relapse rate) in order to explain the observed effect, i.e., to “push” the observed rate ratio to 0.95, i.e., “negligible difference”. It is, therefore, fair to state that the observed estimate of the superior prophylactic efficacy of nitrofurantoin-based treatment vs. other-based treatments when index episode was caused by *E. coli, E. faecalis*, or *E. coli* ESBL is fairly resistant to residual confounding.

### 2.4. Tolerability of the Continuous Antibiotic Prophylaxis

With the limitation of referring practically exclusively to spontaneously reported adverse events over variably long period of treatment, antibiotic prophylaxis was apparently well tolerated—26 women overall (3.0%) reported gastrointestinal disturbances, whereas all other adverse events were sporadic and their relationship to treatment was uncertain (Table 5).

## 3. Discussion

Based on a reasonably large cohort of consecutive women, the present analysis illustrates epidemiological, microbiological, and management characteristics of rUTIs in daily practice, specifically in premenopausal and postmenopausal women, and points out areas for further research. The findings highlight distinct patterns in antibiotic usage, relapse rates, and tolerability, while also underscoring the complexities of various prophylactic strategies.

### 3.1. Antibiotic Prophylaxis Strategies and Their Application

Continuous antibiotic prophylaxis (CAP) was the predominant initial strategy employed in the present cohort, consistent with current guidelines recognizing antibiotics as the most effective method for rUTI prevention [4,5,28,29]. Similar findings in studies by other authors [37,38,39] also emphasize CAP as a primary strategy for rUTI prevention. Nitrofurantoin was the most frequently prescribed antibiotic for CAP in both premenopausal and postmenopausal women, which is in agreement with its established efficacy against common uropathogens like *E. coli* as well as with the current guideline recommendations [40]. This suggests that the initial choice of the “main” antibiotic is rationally guided by the causative agent of the preceding rUTI episode, which is in alignment with a growing emphasis on judicious antibiotic use and accurate diagnosis through urine cultures to guide treatment [41].

However, the subsequent “additions” of postcoital prophylaxis or self-treatment, or the combination with non-antibiotic treatments, appear to be less standardized and more influenced by individual patient factors. Our data indicate an inclination towards flexible or combined approaches, particularly in premenopausal women, with half of them also using additional postcoital prophylaxis. Similarly, a significant proportion of both patient subsets utilized additional non-antimicrobial measures. This variability suggests that these supplementary strategies are largely shaped by individual patient factors, such as personal preferences, rather than strict clinical protocols. The literature acknowledges the existence of various antibiotic prophylaxis regimens but notes that “preferable antibiotic choices are poorly characterized” [39]. This further supports our observation that the integration of diverse prophylactic elements may often be individualized based on patient characteristics and preferences, highlighting a need for clearer guidelines on when and how to integrate these varied prophylactic elements for optimal patient-centered care. Studies often focus on comparing antibiotic prophylaxis to non-antibiotic measures or different antibiotic types rather than on the complex interplay of combined strategies seen in real-world practice [42].

### 3.2. Relapse Rates and Causative Agents

To quantify the occurrence of rUTI relapses, we used event (relapse) rates expressed per 100 patient-months (p-m), as this matric accounts for a variable duration of prophylaxis (as typically seen—for a variety of reasons—in daily practice) and mitigates (as compared to simple proportions or relapsing women) biases arising from unequal exposure times. The present data suggest (at least numerically, based on crude event rates) that relapses seem to be more common in postmenopausal (5.53 relapses/100 p-m) than in premenopausal women (3.14 relapses/100 p-m). This disparity was apparent across different prophylactic modalities, with postmenopausal women consistently demonstrating higher relapse rates. These findings are consistent with the general understanding that postmenopausal women, due to factors like estrogen deficiency and increased prevalence of predisposing conditions, often face a higher burden of rUTIs. Retrospective studies have also highlighted the impact of patient characteristics on prophylaxis effectiveness, with some suggesting differing UTI frequencies between patients meeting and exceeding minimum recurrence thresholds [38].

The most common causative agents identified in relapse episodes for both pre- and postmenopausal women were largely consistent with those of the index episodes, with *E. coli* being overwhelmingly predominant, followed by *K. pneumoniae* and *E. faecalis*. This recurrence of the same primary pathogens in relapse suggests that while prophylaxis is employed, it may not always fully eradicate or prevent re-colonization by these specific, persistent organisms. The presence of *E. coli* ESBL in relapse episodes of postmenopausal women (8/222), and its absence in premenopausal women’s relapses, warrants further investigation into resistance patterns and their implications for prophylactic choices in different age groups. The literature consistently identifies *E. coli* as the primary cause of UTIs and rUTIs [43], reinforcing the importance of empirical and prophylactic strategies targeting this pathogen.

### 3.3. Comparative Efficacy of Nitrofurantoin in Prophylaxis

The complexity of CAP modalities (continuous only, or combined with non-antibiotic options, or continuous + intermittent commonly combined with non-antibiotic options), variety of preferrable antibiotics, and the index episode causative agents (that guided the choice of prophylactic treatments) in the present cohort was such that a meaningful comparison between modalities or between patient subsets (post- or premenopausal) appeared practically impossible. On the other hand, the reported cohort enabled the assessment of comparative efficacy of nitrofurantoin-based CAP vs. CAP based on other antibiotics (predominantly fosfomycin) in women whose index rUTI episodes were caused by *E. coli*, *E. faecalis*, or *E. coli* ESBL, as these are the pathogens for which nitrofurantoin is a legitimate prophylactic option. This targeted comparison was essential because the efficacy of prophylactic antibiotics can only be reasonably assessed against similar causative agents and in patient populations in which at least some control of potential confounding variables is possible. By employing optimization-based weighting for postmenopausal women and exact matching for premenopausal women, we controlled for several important covariates, such as age, prophylactic modality, and number of risk factors. The results indicate a substantially lower relapse rates for nitrofurantoin-based prophylaxis compared to fosfomycin or other antibiotic-based prophylaxis. Specifically, the adjusted rate ratio (nitrofurantoin vs. fosfomycin/other) was 0.47 (95% CI 0.34–0.67) in postmenopausal women, and 0.35 (95% CI 0.08–1.46) in premenopausal women. While the premenopausal estimate is less precise due to smaller sample sizes, the trend mirrors that of postmenopausal women. The E-value for the rate ratio of 0.47 in postmenopausal women indicates that a substantial and highly improbable level of unmeasured confounding (requiring a hypothetical confounder to be 3.46 times more prevalent in nitrofurantoin-treated women and have a strong prophylactic effect) would be required to entirely explain the observed protective effect of nitrofurantoin. This strongly suggests that for rUTIs primarily driven by *E. coli*, *E. faecalis*, or *E. coli* ESBL, nitrofurantoin likely represents a genuinely more effective prophylactic choice compared to the alternative regimens studied.

When comparing our findings with the existing literature on the prophylactic efficacy of fosfomycin and nitrofurantoin, we find some points of convergence as well as of divergence. A systematic review and meta-analysis by Jent et al. focusing on prevention of recurrent UTIs found that in nine head-to-head trials, “the efficacy of the antibiotic agents appeared similar: the pooled RR indicated no difference between nitrofurantoin and comparators (RR, 1.01; 95% CI, 0.74–1.37)” [39]. This contrasts with the present findings of superior efficacy for nitrofurantoin in specific pathogen contexts. Another systematic review by Price et al. also compared nitrofurantoin with other agents for reducing recurrent UTIs [25]. While many studies compared these antibiotics for treatment of uncomplicated UTIs, generally showing similar or slightly better efficacy for nitrofurantoin [40,44,45,46,47], direct comparative studies in prophylaxis are less abundant and sometimes present mixed results. For instance, Rudenko and Dorofeyev conducted a placebo-controlled study evaluating fosfomycin trometamol for the prevention of infectious recurrences of lower urinary tract [36]. The discrepancy between our findings and some of the meta-analyses may stem from differences in study design, specific patient populations (e.g., menopausal status), the particular pathogens included, and biases more likely to occur in non-randomized settings (like the present one) than in randomized trials. However, we believe that the present analysis achieved a fair level of “bias-protection” and indicated a strong effect of nitrofurantoin not likely to be completely explained by systematic errors. Moreover, it reflected a real-world setting, focusing on pathogen-specific efficacy, and thus provided valuable insights that complement broader meta-analyses of randomized trials.

It is crucial to acknowledge certain limitations in this comparison. While important covariates were addressed, factors such as the use of systemic or topical hormone replacement therapy and the precise duration of antibiotic exposure were not fully controlled. These unmeasured variables could introduce residual confounding. Furthermore, our findings regarding the superior efficacy of nitrofurantoin for the specified pathogens cannot be generalized to rUTIs caused by other bacteria, such as *P. mirabilis* or *K. pneumoniae*, for which different classes of antibiotics (e.g., quinolones, trimethoprim–sulfamethoxazole) are typically selected for prophylaxis. Guidelines emphasize that empirical antibiotic choice should be guided by local resistance patterns and the specific pathogen [40,48], and such an approach was practiced in the reported cohort.

### 3.4. Tolerability of Antibiotic Prophylaxis

Overall, continuous antibiotic prophylaxis in the present cohort appeared well tolerated, with only 3.0% of all women reporting gastrointestinal disturbances, and other adverse events being sporadic. Interestingly, premenopausal women reported a higher incidence of gastrointestinal (8.4% vs. 0.9%), general (5.0% vs. 0.6%), and hepatological (5.0% vs. 0.3%) adverse events compared to postmenopausal women. This difference might have been influenced by various factors, including underlying health status, concurrent medications, or differences in reporting patterns between the age groups. The literature indicates that antibiotic prophylaxis may be associated with gastrointestinal and other side effects, including *C. difficile* infection, and the development of antimicrobial resistance [38]. While our study noted *C. difficile* diarrhea in a small number of postmenopausal women, the overall incidence of severe adverse events remained low. The limitation of relying on spontaneously reported adverse events over variable treatment durations means that the true incidence and spectrum of side effects might have been underestimated, particularly for milder or subclinical reactions. However, the data suggest that while nitrofurantoin shows promising efficacy for specific pathogens, careful monitoring for adverse effects, especially gastrointestinal and hepatological, remains important, particularly in premenopausal women.

### 3.5. Recommendations According to Our Clinical Practice

Due to the numerous clinical considerations regarding patients with recurrent urinary tract infections (rUTIs), it is recommended to evaluate the following before initiating antimicrobial prophylaxis:

1. Urine Culture Isolate
  (a) *Sensitivity of the isolated bacteria to antibiotics that can be used in prophylaxis*: If the causative agent is sensitive only to broad-spectrum antibiotics, the necessity of antimicrobial prophylaxis should be reassessed. If the potential for side effects outweighs the benefit to the patient, it may be better to treat each episode individually.
  (b) *Occurrence of different isolates in urine cultures*: This refers to the (in)feasibility of using a single antimicrobial drug for prophylaxis. If antimicrobial prophylaxis is ineffective against all isolates appearing in repeated urine cultures in rUTIs, there is a high likelihood of recurrence caused by a uropathogen not covered by the prophylactic antibiotic. Although antimicrobial prophylaxis is usually based on the most recent positive urine culture, when selecting an antimicrobial agent for patients with multiple different isolates in their urine cultures, it is ideal to choose an antibiotic that is effective against all isolates.
2. Choice of Antibiotic

Choose the narrowest-spectrum antibiotic that is effective against uropathogen isolated in urine culture and that minimally impacts intestinal and vaginal flora (e.g., nitrofurantoin, nitroxoline) and has the fewest potential side effects (e.g., fosfomycin).

3. Patient Characteristics
  (a) *Age*: Verify whether the antibiotic intended for prophylactic use is associated with a higher risk of side effects in older adults, e.g., nitrofurantoin (refer to “Beers criteria for inappropriate medication use in older patients” [49]). For women of childbearing age, select antibiotics that are safe during pregnancy.
  (b) *Chronic illnesses and ongoing treatment*: Consider possible interactions between the patient’s current therapy and the antibiotic intended for prophylaxis.
  (c) *Renal and liver function*: Especially in older patients, check serum creatinine, creatinine clearance, and liver enzymes before starting prophylaxis. Some antibiotics have usage restrictions in cases of renal or hepatic insufficiency (e.g., nitrofurantoin), and antibiotics themselves may worsen pre-existing kidney or liver damage.
  (d) *Antibiotic allergies*
4. Possible Complications/Side Effects of Antibiotics

This includes direct toxicity, selection of resistant microorganisms, *Clostridioides difficile* diarrhea, vulvovaginal candidiasis, etc. All side effects must be noted at regular check-ups.

5. Expectations

One should expect disappearance or relief of UTI symptoms, otherwise the prophylaxis is considered ineffective. Even though we usually perform urine culture during antimicrobial prophylaxis, positive urine culture is considered as asymptomatic bacteriuria if it is not associated with symptoms of UTI. However, a change in antimicrobial agent in patients must be considered due to the possibility of recurrences caused by uropathogen that is not sensitive to antimicrobial use in prophylaxis. Patients should be warned that rUTIs may recur once long-term antimicrobial prophylaxis is discontinued.

6. Predisposing Factors

Identify predisposing factors for potential removal. rUTIs in the presence of functional (e.g., urinary incontinence, overactive bladder, urine retention) or structural (e.g., prolapsed uterus, cystocele, bladder diverticulum, urinary catheter) abnormalities of the urinary tract often require long-term antimicrobial prophylaxis if these factors cannot be eliminated.

Therefore, in older patients, at the minimum, an abdominal and urinary system ultrasound with post-void residual urine assessment is indicated. In some cases, consultation with other specialists (e.g., urologist, gynecologist) is required, depending on the functional or structural abnormalities found.

Postmenopausal women should routinely be referred for a gynecological examination for two reasons: firstly, to assess the risk associated with the use of local estrogen therapy and initiate its prescription; and secondly, to evaluate possible anatomical abnormalities of the genital tract which may contribute to the development of rUTIs. Women with urinary incontinence should be referred to a urogynecologist or a urodynamic specialist in order to evaluate possible treatment modalities which may also contribute to a reduction in the frequency of rUTIs.

Another aspect not mentioned in the guidelines is to monitor patients who are on antimicrobial prophylaxis. Based on the long-standing tradition of our Outpatient Clinic for Urogenital Infections and advice from experienced colleagues, we typically conduct regular check-ups every three to four months, which include the following:

1. Clinical Evaluation—assessment of how well the patient tolerates antimicrobial prophylaxis, presence or absence of UTI symptoms, and documentation of side effects.
2. Laboratory Tests
  (a) *Urinalysis and urine culture* (collected without interrupting prophylaxis)—urinalysis should not indicate inflammation, and the culture is preferably sterile.
  (b) *Complete blood count*—monitor for anemia, leukopenia and neutropenia (with nitrofurantoin), and eosinophilia (possible with all antibiotics).
  (c) *Serum creatinine*—should be within reference range for certain antibiotics (e.g., nitrofurantoin).
  (d) *Liver function tests*—abnormal results may be due to antibiotic use.
3. Reassessment of the need for continued prophylaxis or a shift to a different prophylactic strategy (e.g., postcoital prophylaxis, self-treatment, or non-antimicrobial prophylaxis), depending on the patient’s clinical status and preferences.

This approach has proven effective, as it allows for the monitoring of antibiotic side effects, UTI recurrences, and the patient’s overall condition.

## 4. Materials and Methods

We conducted an anonymized retrospective review of the medical records of consecutive adult women suffering from recurrent urinary tract infections (rUTIs) managed at the Outpatient Clinic for Urogenital Infections, University Hospital for Infectious Diseases “Dr. Fran Mihaljević” in Zagreb, Croatia between 1 January 2022 and 30 December 2024.

### 4.1. Patients

Eligible for inclusion were (i) adult women (age >18 years) with verified recurrent urinary tract infection (rUTI) defined as two or more infections within the past 6 months, or three or more infections within the past year, that were confirmed by urine culture, and in which antimicrobial prophylaxis was indicated (the ICD-10 codes for all included women were N30.20, N30.80, N30.90, or Z87.440); (ii) women with a minimum observational period of 6 months after the first visit, i.e., patients who had their first visit to the Outpatient clinic at the end of 2024 and were followed-up till 15 July 2025; (iii) women with at least one control visit after the first visit. Patients who did not return for check-ups in person were contacted by phone or by e-mail with an inquiry about UTI symptoms, possible recurrences, and adverse events. Patients with symptoms of UTI that were suspected to be caused by other underlying diseases, those without urinary culture confirmation of a causative agent in the index episode, and a few patients who could not be followed up by any means were not included in the present analysis.

### 4.2. Data Sources and Management

For the present analysis, patients were identified. and data were retrieved from the Institution’s electronic database. The actual patient data were entered into the database in real-time (i.e., prospectively) at the first and all subsequent visits by the staff of the Outpatient Clinic specialized in the management of urogenital infections, following a standardize template: demographics, habits and lifestyle, medical histories, comorbidities, comedication, specific (r)UTI treatment (acute episode and prophylaxis) (active principle, posology, complementary treatments), clinical and laboratory (microbiology, chemistry–biochemistry) findings, disease recurrence, treatment tolerability, and adverse events (typically—self-reported). Data of interest were extracted into a standardized Excel sheet, and the source and the extracted data were checked twice.

### 4.3. Data Analysis

The main objective was to provide a detailed description (summary statistics) of microbiological and treatment characteristics (in graphical or tabulated format) pertinent to the acute UTI episode (index episode) and the subsequent prophylactic treatment and occurrence of rUTI relapses (while on treatment) separately in postmenopausal and in premenopausal women. Having in mind—and based on previous experience—the expected range of different prophylactic practices, we could not a priori assume that any inferential analysis would be possible. However, upon data review, it appeared plausible to estimate relative efficacy of nitrofurantoin-based prophylactic treatments compared to other antibiotic-based prophylactic treatments in subjects with common causative agents identified in the index episode. Since the patient subsets of interest were relatively limited in size, we considered “classical” multivariable regression models infeasible (too few relapse events, too many covariates to account for). We, therefore, attempted to achieve balance between compared patient (treatment) subsets on relevant covariates using optimization-based weighting [50], [implemented in package *WeighIt* [51] in R language and environment for statistical computing, R foundation for Statistical computing] or exact matching [implemented in package *MatchIt* [52] in R], whichever enabled better trade-off in the achieved covariate balance between the compared patient subsets and increase in variance. Balanced data were analyzed by fitting a (weighted) Poisson model with log(duration of prophylactic treatment in days) as the offset to generate relapse rates per 100 patient-months of exposure and rate ratios with 95% confidence intervals. We used SAS for Windows 9.4 software (SAS Inc., Cary, NC, USA). Finally, to estimate susceptibility of the generated estimates to residual confounding, we calculated the E-value (indicates strength of association that a potential unmeasured confounder needs to have with both the exposure and the outcome in order to completely explain the observed effect) [53].

## 5. Conclusions and Limitations

This study highlights distinct patterns in rUTI prophylaxis and outcomes between premenopausal and postmenopausal women. CAP, predominantly with nitrofurantoin, remains the primary strategy with supplementary therapies most often reflecting individualized patient choices. Postmenopausal women consistently exhibit higher relapse rates, reinforcing the impact of age-related factors. Our analysis demonstrates that nitrofurantoin-based prophylaxis significantly reduces relapse rates compared to fosfomycin or other antibiotics for rUTIs caused by *E. coli*, *E. faecalis*, or *E. coli* ESBL, particularly in postmenopausal women. While antimicrobial prophylaxis is generally well-tolerated, adverse event profiles vary with premenopausal women reporting higher incidences than postmenopausal women. These findings underscore nitrofurantoin’s role as a preferred prophylactic option for specific uropathogens and emphasize the need for individualized rUTI management approaches, acknowledging differences among women of varying menopausal statuses.

The present analysis is based on routinely collected data with variable patient follow-up during prophylactic treatment and treatment (non)-compliance of unknown extent. On the other hand, the data that were captured prospectively in real-time by the staff specialized in urinary tract infections, involved each patient having at least two visits during the prophylactic treatment. Under these conditions, the captured data could be reasonably considered reliable, but some of the events of interest (e.g., relapses, adverse events) were likely missed. Under these circumstances, it appears fair to state that the data adequately describe the daily practice of rUTI management in post- and premenopausal women. With the same limitations in mind—of which lack of data on hormonal replacement therapy in postmenopausal women is particularly important—it still seems fair to conclude that the data suggest that in both of these subsets of women with rUTI, nitrofurantoin-based treatment might be the preferable prophylactic option compared to other antibiotics when the previous acute UTI episode was caused by *E. coli*, *E. faecalis*, or *E. coli* ESBL.

## Figures and Tables

**Table 1:** Characteristics of peri-/postmenopausal women who received continuous antibiotic prophylaxis: only continuous antibiotic (continuous only); combined with non-antibiotic treatments (continuous + non-atb); or a combination of continuous prophylaxis and intermittent additional antibiotic use (postcoital or self-management), which might have also included non-antibiotic treatments (continuous + intermittent). Data are mean ± standard deviation (range) or median (range) for prophylaxis duration, mean (95% CI) for relapse rates, or count (percent).

	All	Continuous Only	Continuous + Non-atb	Continuous + Intermittent
N	630	476	97	57
Age (years)	68 ± 9 (45–89)	68 ± 9 (45–89)	67 ± 10 (45–87)	64 ± 10 (48–88)
Urine incontinence	220 (34.9)	182 (38.2)	22 (22.7)	16 (28.1)
Bladder diverticulosis	9 (1.4)	9 (1.9)	0	0
Urine retention	25 (4.0)	21 (4.4)	3 (3.1)	1 (1.7)
Self-catheterization	7 (1.1)	5 (1.1)	1 (1.0)	1 (1.7)
Urogenital prolapse	25 (4.0)	20 (4.2)	2 (2.1)	3 (5.3)
Diabetes mellitus	84 (13.3)	69 (14.5)	12 (12.4)	3 (5.3)
Urolithiasis	45 (7.1)	34 (7.1)	10 (10.3)	1 (1.7)
Hydronephrosis	8 (1.3)	7 (1.5)	1 (1.0)	0
Chronic kidney disease	18 (2.9)	13 (2.7)	3 (3.1)	2 (3.5)
Bladder prosthesis	2 (0.3)	2 (0.4)	0	0
Cystocele	66 (10.5)	56 (11.8)	5 (5.2)	5 (8.8)
Rectocele	13 (2.1)	13 (2.7)	0	0
Urogenital surgery	4 (0.6)	3 (0.6)	1 (1.0)	0
Total risk factors	1 (0–4)	1 (0–2)	0 (0–2)	0 (0–2)
0	265 (42.1)	181 (38.0)	50 (51.6)	34 (59.7)
1	233 (37.0)	183 (38.5)	35 (36.1)	15 (26.3)
≥2	132 (20.9)	112 (23.5)	12 (12.4)	8 (14.0)
Prophylaxis (months)	6 (0.25–36.0)	6 (0.25–36.0)	6 (0.5–18)	6 (0.5–36)
Total patient-months	4011	3057.5	569.5	384
Relapse	222 (35.2)	166 (34.9)	41 (42.3)	15 (26.3)
Relapse rate/100 p-m	5.54 (4.88–6.28)	5.43 (4.68–6.30)	7.20 (5.58–9.28)	3.91 (2.34–6.52)
Agents in relapse				
*E. coli*	132/222 (60.0)	97/166 (58.4)	26/41 (63.4)	9/15 (60.0)
*K. pneumoniae*	27/222 (12.3)	20/166 (12.0)	4/41 (9.8)	3/15 (13.3)
*E. faecalis*	15/222 (6.8)	10/166 (6.0)	3/41 (7.3)	2/15 (13.3)
*P. mirabilis*	12/222 (5.5)	10/166 (6.0)	1/41 (2.4)	1/15 (6.7)
*E. coli* ESBL	8/222 (3.6)	8/166 (4.8)	0/41	0/15
*P. aeruginosa*	5/222 (2.3)	4/166 (2.4)	1/41 (2.4)	0/15
*E. cloacae*	3/222 (1.4)	2/166 (1.2)	1/41 (2.4)	0/15
*K. aerogenes*	3/222 (1.4)	3/166 (1.8)	0/41	0/15
*K. pneumoniae* ESBL	3/222 (1.4)	2/166 (1.2)	1/41 (2.4)	0/15
*C. freundii*	2/222 (0.9)	2/166 (1.2)	0/41	0/15
*C. koseri*	2/222 (0.9)	1/166 (0.6)	1/41 (2.4)	0/15
*K. oxytoca*	2/222 (0.9)	1/166 (0.6)	1/41 (2.4)	0/15
*M. morganii*	2/222 (0.9)	2/166 (1.2)	0/41	0/15
*Acinetobacter* spp.	1/222 (0.45)	1/166 (0.6)	0/41	0/15
*Citrobacter* spp.	1/222 (0.45)	1/166 (0.6)	1 (2.4)	0/15
*K. oxytoca* ESBL	1/222 (0.45)	1/166 (0.6)	0/41	0/15
*S. marcenscens*	1/222 (0.45)	0/166	1/41 (2.4)	0/15
Unknown	0/222	2/166 (1.2)	0/41	0/15

**Table 2:** Characteristics of premenopausal women who received continuous antibiotic prophylaxis: only continuous antibiotic (continuous only), or combined with non-antibiotic treatments (continuous + non-atb), or a combination of continuous prophylaxis and intermittent additional antibiotic use (postcoital or self-management), which might have also included non-antibiotic treatments (continuous + intermittent). Data are mean ± standard deviation (range) or median (range) for prophylaxis duration, mean (95% CI) for relapse rates, or count (percent).

	All	Continuous Only	Continuous + Non-atb	Continuous + Intermittent
N	238	104	21	113
Age (years)	34 ± 8 (18–51)	36 ± 8 (18–51)	38 ± 7 (24–49)	32 ± 8 (18–47)
Urine incontinence	7 (2.9)	2 (1.9)	3 (14.3)	2 (1.8)
Bladder diverticulosis	0	0	0	0
Urine retention	7 (2.9)	5 (4.8)	1 (4.8)	1 (0.9)
Self-catheterization	3 (1.3)	3 (2.9)	0	0
Urogenital prolapse	1 (0.4)	1 (1.0)	0	0
Diabetes mellitus	1 (0.4)	1 (1.0)	0	0
Urolithiasis	5 (2.1)	4 (3.8)	0	1 (0.9)
Hydronephrosis	1 (0.4)	1	0	0
Chronic kidney disease	0	0	0	0
Bladder prosthesis	1 (0.4)	1 (1.0)	0	0
Cystocele	2 (0.8)	2 (1.9)	0	0
Rectocele	0	0	0	0
Urogenital surgery	0	0	0	0
Pregnancy	10 (4.2)	10 (9.6)	0	0
Total risk factors	0 (0–2)	0 (0–2)	0 (0–1)	0 (0–1)
0	208 (87.4)	82 (78.9)	17 (81.0)	109 (96.5)
1	23 (9.7)	15 (14.4)	4 (19.0)	4 (3.5)
≥2	7 (2.9)	7 (6.7)	0	0
Prophylaxis (months)	6 (0.5–36)	5 (0.5–24)	6 (2–36)	6 (1–18)
Total patient-months	1337	555	180	602
Relapse	42 (17.6)	22 (21.1)	6 (28.6)	14 (12.4)
Relapse rate/100 p-m	3.14 (2.34–4.21)	3.96 (2.59–6.07)	3.33 (1.74–6.38)	2.33 (1.40–3.871)
Agents in relapse				
*E. coli*	26/42 (61.9)	10/22 (45.5)	5/6	11/14 (78.6)
*K. pneumoniae*	3/42 (7.1)	3/22 (13.6)	0/6	0/14
*E. faecalis*	4/42 (9.5)	3/22 (13.6)	1/6	0/14
*P. mirabilis*	2/42 (4.8)	1/22 (4.6)	0/6	1/14 (7.1)
*P. aeruginosa*	1/42 (2.4)	1/22 (4.6)	0/6	0/14
*C. koseri*	1/42 (2.4)	0/22	0/6	1/14 (7.1)
*K. oxytoca*	1/42 (2.4)	0/22	0/6	1/14 (7.1)
BHS-B	2/42 (4.8)	2/22 (9.1)	0/6	0/14
*E. faecium*	1/42 (2.4)	1/22 (4.6)	0/6	0/14
*S. saprophyticus*	1/42 (2.4)	1/22 (4.6)	0/6	0/14

BHS-B—Beta-Haemolytic Streptococcus group B.

**Table 3:** Characteristics of post- and premenopausal women in whom the index recurrent urinary tract infection episode was caused by *E. coli, E. coli* ESBL or *E. faecalis*, for which nitrofurantoin is a legitimate option according to the received prophylactic treatment: treatment based on nitrofurantoin or treatment based on fosfomycin (predominantly) or other antibiotics (depicted in Figure 4). Treatment could be continuous, continuous + intermittent combined with any antibiotic, with or without additional non-antibiotic treatments. Data are mean ± standard deviation (range) or median (range) for prophylaxis duration, mean (95% CI) for relapse rates, or count (percent).

	Postmenopausal Women		Premenopausal Women	
	Nitrofurantoin	Fosfomycin or Other	Nitrofurantoin	Fosfomycin or Other
N	425	111 (91 + 20)	185	26 (16 + 10)
Age (years)	67 ± 9 (45–88)	71 ± 10 (49–89)	34 ± 8 (18–51)	36 ± 7 (23–48)
Causative index agents				
*E. coli*	379 (89.2)	99 (89.2)	168 (90.8)	24 (92.3)
*E. faecalis*	25 (5.9)	9 (8.1)	14 (7.6)	2 (7.7)
*E. coli* ESBL	21 (4.9)	3 (2.7)	3 (1.6)	0
Total risk factors	1 (0–4)	1 (0–4)	0 (0–2)	0 (0–3)
0	193 (45.4)	39 (35.1)	166 (89.7)	18 (69.2)
1	152 (35.8)	46 (41.4)	15 (8.2)	6 (23.1)
≥2	80 (18.8)	26 (23.4)	4 (2.2)	2 (7.7)
Only continuous therapy	384 (90.3)	100 (90.1)	86 (46.5)	20 (76.9)
Continuous + intermittent	41 (9.7)	11 (9.9)	99 (53.5)	6 (23.1)
Additional non-antibiotic	81 8 (19.1)	27 (24.3)	80 (43.2)	5 (19.1)
Prophylaxis (months)	6 (0.5–36.0)	4 (1.0–19.0)	6 (0.5–36.0)	4 (1.0–12.0)
Total patient-months	3010.25	561.50	1100.5	113
Relapse	131 (30.8)	56 (51.4)	29 (15.7)	6 (23.1)
Relapse rate/100 p-m	4.35 (3.69–5.14)	10.2 (8.12–12.7)	2.36 (1.86–3.73)	5.31 (2.39–11.8)
Agents in relapse				
*E. coli*	85/131 (65.4)	28/56(50.0)	17/29 (58.6)	4/6
*K. pneumoniae*	13/131 (10.0)	10/56 (17.9)	2/29 (6.9)	0/6
*P. mirabilis*	9/131 (6.9)	1/56 (1.8)	2/29 (6.9)	0/6
*E. faecalis*	8/131 (6.2)	6/56 (10.7)	2/29 (6.9)	1/6
*P. aeruginosa*	3/131 (2.3)	0/56	1/29 (3.4)	0/6
*K. aerogenes*	3/131 (2.3)	0/56	0/29	0/6
*E. coli* ESBL	2/131 (1.5)	5/56 (8.9)	0/29	0/6
*K. oxytoca*	0/131	2/56 (3.6)	1/29 (3.4)	0/6
*K. oxytoca* ESBL	0/131	1/56 (1.8)	0/29	0/6
*K. pneumoniae* ESBL	1/131 (0.8)	1/56 (1.8)	0/29	0/6
*E. cloacae*	1/131 (0.8)	1/56 (1.8)	0/29	0/6
*Acinetobacter* spp.	1/131 (0.8)	0/56	0/29	0/6
*Citrobacter* spp.	0/131	1/56 (1.8)	0/29	0/6
*C. freundii*	1/131 (0.8)	0/56	0/29	0/6
*C. koseri*	1/131 (0.8)	0/56	0/29	1/6
*M. morganii*	1/131 (0.8)	0/56	0/29	0/6
*S. marcescens*	1/131 (0.8)	0/56	0/29	0/6
BHS-B	0/131	0/56	2/29 (6.9)	0/6
*E. faecium*	0/131	0/56	1/29 (3.4)	0/6
*S. saprophyticus*	0/131	0/56	1/29 (3.4)	0/6

BHS-B—Beta-Haemolytic Streptococcus group B.

**Table 4:** Estimated adjusted relapse rates and rate ratios for nitrofurantoin-based vs. fosfomycin or other antibiotic-based prophylaxis in women with index episode caused by *E. coli*, *E. faecalis*, or *E. coli* ESBL. Standardized difference (d) < 0.1 indicates irrelevant differences. Data are weighted mean ± standard deviation or weighted proportions (%).

	Postmenopausal Women ^1^			Premenopausal Women ^2^		
	Nitrofurantoin	Fosfomycin or Other	d	Nitrofurantoin	Fosfomycin or Other	d
N	425	111	---	169 matched	26 matched	---
*Used for weighting/matching*						
Age (years)	67 ± 9	68 ± 10	0.075	33 ± 8	36 ± 9	---
Causative index agents						
*E. coli*	89.2	89.2	0.000	94.9	94.9	0.000
*E. faecalis*	5.2	5.2	0.000	5.1	5.1	0.000
*E. coli* ESBL	5.6	5.6	0.000	0.0	0.0	0.000
Number of risk factors						
0	43.2	43.2	0.000	91.3	91.3	0.000
1	36.9	36.9	0.000	5.6	5.6	0.000
≥2	19.8	19.8	0.000	3.1	3.1	0.000
Only continuous therapy	90.3	90.3	0.000	49.7	49.7	0.000
Continuous + intermittent	9.7	9.7	0.000	50.3	50.3	0.000
Additional non-antibiotic	20.1	20.1	0.000	40.0	40.0	0.000
*Outcomes*						
Relapse rate/100 p-m	4.59 (2.93–7.20)	9.70 (5.91–15.2)	---	2.54 (1.71–3.78)	7.25 (1.81–29.1)	---
Rate ratio (nitrofurantoin vs. fosfomycin/other)	0.47 (0.34–0.67)		---	0.35 (0.08–1.46)		---

^1^ Balance achieved by optimization-based weighting. For patients treated with nitrofurantoin-based prophylaxis: effective sample size 422.13, mean weight = 1.0, mean absolute deviation = 0.066; for patients treated with fosfomycin or other antibiotic-based prophylaxis: effective sample size 95.7, mean weight = 1.0, mean absolute deviation 0.316. ^2^ Balance by exact matching on categorical variables. Age included in the regression model. For patients treated with nitrofurantoin-based prophylaxis (169 matched): effective sample size 163.4, mean weight = 1.0, SD = 0.19; for patients treated with fosfomycin or other antibiotic-based prophylaxis (26 matched): effective sample size 13.2, mean weight = 1.0, SD = 1.0.

**Table 5:** Adverse events reported by the women who received antibiotic prophylaxis.

	All Women	Postmenopausal	Premenopausal
N	868	630	238
Gastrointestinal adverse events	26 (3.0)	6 (0.9)	20 (8.4)
General (e.g., “malaise, fatigue, feeling unwell”)	16 (1.8)	4 (0.6)	12 (5.0)
Hepatological (liver enzyme increases)	14 (1.6)	2 (0.3)	12 (5.0)
Hematological (e.g., blood cell counts alterations)	12 (1.4)	5 (0.8)	7 (2.8)
Hypersensitivity reactions (e.g., skin rashes)	10 (1.2)	4 (0.6)	6 (2.5)
Neurological (e.g., dizziness, headache)	7 (0.8)	2 (0.3)	5 (2.1)
Pulmonary (e.g., cough)	5 (0.6)	3 (0.5)	3 (1.3)
*C. difficile* diarrhea	2 (0.2)	2 (0.3)	0
Vulvovaginal candidiasis	2 (0.2)	0	2 (0.8)
Other	4 (0.5)	0	4 (1.7)

## Data Availability

This research used institutional data that is not available for sharing.
